# Prospects of Replication-Deficient Adenovirus Based Vaccine Development against SARS-CoV-2

**DOI:** 10.3390/vaccines8020293

**Published:** 2020-06-10

**Authors:** Mariangela Garofalo, Monika Staniszewska, Stefano Salmaso, Paolo Caliceti, Katarzyna Wanda Pancer, Magdalena Wieczorek, Lukasz Kuryk

**Affiliations:** 1 Department of Pharmaceutical and Pharmacological Sciences, University of Padova, Via F. Marzolo 5, 35131 Padova, Italy; stefano.salmaso@unipd.it (S.S.); paolo.caliceti@unipd.it (P.C.); 2 Chair of Drug and Cosmetics Biotechnology, Faculty of Chemistry, Warsaw University of Technology, Noakowskiego 3, 00-664 Warsaw, Poland; mstaniszewska@ch.pw.edu.pl; 3 Department of Virology, National Institute of Public Health—National Institute of Hygiene, Chocimska 24, 00-791 Warsaw, Poland; kpancer@pzh.gov.pl (K.W.P.); mrechnio@pzh.gov.pl (M.W.); 4 Clinical Science, Targovax Oy, Saukonpaadenranta 2, 00180 Helsinki, Finland

**Keywords:** SARS-CoV-2, vaccine, adjuvant, adenovirus, COVID-19

## Abstract

The current appearance of the new SARS coronavirus 2 (SARS-CoV-2) and it quickly spreading across the world poses a global health emergency. The serious outbreak position is affecting people worldwide and requires rapid measures to be taken by healthcare systems and governments. Vaccinations represent the most effective strategy to prevent the epidemic of the virus and to further reduce morbidity and mortality with long-lasting effects. Nevertheless, currently there are no licensed vaccines for the novel coronaviruses. Researchers and clinicians from all over the world are advancing the development of a vaccine against novel human SARS-CoV-2 using various approaches. Herein, we aim to present and discuss the progress and prospects in the field of vaccine research towards SARS-CoV-2 using adenovirus (AdV) replication deficient-based strategies, with a comprehension that may support research and combat this recent world health emergency.

## 1. Introduction

In December 2019, the World Health Organization (WHO) reported a cluster of pneumonia cases of unknown origin registered in Wuhan, Hubei Province, China [1]. Most of these cases were epidemiologically connected to the Huanan Seafood Wholesale Market, probably related to animal contact. Subsequently, the first incidence of human-to-human transmission occurred [2] and the disease, now named coronavirus disease 19 (COVID-19), had a rapid and global dissemination. The emerging pathogen was detected and rapidly characterized as a new member of the beta-coronavirus genus, nearly associated to several bat coronaviruses and to severe acute respiratory syndrome coronavirus (SARS-CoV) [3]. The whole genome sequencing highlighted that SARS coronavirus 2 (SARS-CoV-2) has in common a sequence homology with SARS-CoV, within the receptor-binding motif that precisely interacts with the mankind receptor Angiotensin Converting Enzyme 2 (ACE2). This aspect provides valuable indications for vaccine development. The human-to-human spread of SARS-CoV-2 has now been confirmed, and the World Health Organization (WHO) has declared COVID-19 a health emergency of global concern. Furthermore, the new SARS-coronavirus was found in patients and is considered to be the causative agent of the new emerging lung disease [1], acute kidney injury (AKI) [4,5], ischemia stroke and blood clots [5,6], and affects the nervous, vascular, digestive, urinary and haematological systems [7]. Therefore, this recent issue poses a global health emergency.

Vaccines are an important and effective public health tool, able to contribute to the prevention and control of infectious diseases that can cause serious, even fatal, complications [8] representing a safe strategy compared to therapeutic medicines [9]. Despite tremendous global efforts to contain SARS-CoV-2, the virus continues to spread around the world. Therefore, the development of novel and effective vaccines for SARS-CoV-2 are urgently warranted, since some natural infections may not result in long-lasting protective immunity. The disease-induced immunity is often induced by a single natural infection, while an immunity caused by vaccine administration usually occurs after few administrations. Nevertheless, immunization with vaccines, in contrast to some natural infections, induces long-lasting protecting immunity [10].

Herein, we will focus on the use of adenoviruses as attractive vaccine adjuvants and vectors, highlighting the clinical prospects on vaccine generation against SARS-CoV-2. We hope that this brief review will support the global community and help in designing the appropriate immune intervention for the prophylactic vaccines against SARS-CoV-2.

## 2. Vaccines as a Tool to Prevent Infectious Diseases

Vaccination is one of the most meaningful achievements of medicine that has enormously limited the spread of infectious diseases over the last 20 decades [11,12]. Discovered in the 1700s, vaccination is considered a useful economic strategy able to preserve human health by preventing or eradicating infectious diseases, thus helping to maintain a high quality of life [13]. During the 20th century, mass vaccination led to a huge decrease in the incidence and morbidity of many infectious diseases and, interestingly, in various countries vaccination became a routine strategy that led to the reduction and elimination of a number of infectious diseases [12]. Nevertheless, while the smallpox vaccine represents a successful vaccine achievement which lead to the disease being eradicated globally, there is still the urgency to develop safe and cost-effective vaccines against recent diseases such as HIV and SARS-CoV-2, which are currently severe issues for public health worldwide.

In addition to directly preventing diseases, vaccinations can also reduce disease transmission. In fact, due to the herd immunity, persons who do not receive a vaccination have a reduced risk of being infected once a high percentage of the population has been immunized [14]. Moreover, vaccination can also limit the use of antibiotic based therapy [15,16], thus affecting the antimicrobial resistance, as proved [12]. Interestingly, being effective in reducing disease currency and associated mortality, vaccination also avoids additional costs of medical care.

Despite this, there is still a need for long-lasting vaccine platforms. Therefore, developing better ways to prevent and manage pandemic threats will be critical to control infectious diseases.

## 3. SARS-CoV-2 Structure and Protein Composition

SARS-CoV-2 is close enough to both Middle East respiratory syndrome-related coronavirus (MERS-CoV) and SARS-CoV, and it displays features typical of betacoronaviruses as follows: envelope and positive single-stranded ss-RNA (29,903 bp) [16,17,18,19,20]. The RNA organisational genome structure, starting from 5′ to 3′, uncovers the following: 5′-untranslated-region (UTR), replicase complex (ORF1a and ORF1b), four structural proteins (S,E,M,N)-3′, and non-structural ORFs [21,22]. ORF1a and ORF1b encompass about two-thirds of the genome [2]. According to the authors [23,24], ORF1a and ORF1b undergo frameshift mutations resulting in both pp1a (440–500 kDa) and pp1ab (740–810 kDa) non-structural polypeptides (nsps). These nsps are operated by viral proteases: chymotrypsin (3CLpro), main Mpro, and two papain producing sixteen nsps [23,25,26]. The SARS-CoV nsps are dispensable for virus replication (nsp12 harbouring RNA-dependent RNA polymerase RdRP) and blocking the host innate immune response [14]. The varial folding and RNA synthesis depend on the specific proteins M, E and N, respectively [27,28]. Interestingly, SARS-2-S and SARS-S show the following similarities: (1) amino acids (~76% identity) [15,21,27,29]; (2) entry into the host cells using two receptors (angiotensin-converting enzyme 2-ACE2 and cellular serine protease-TMPRSS2) and (3) priming spike-protein (S protein) [27,30]. There were found [17,23] to be at least 41 modifications in SARS-CoV-2’s transcript [5], with the most frequent motive of AAGAA [25]. The SARS-CoV-2’s genomic and proteomic sequences are published in GISAID, NCBI, NMDC and CNCB/NGDC. To date, it was shown that SARS-CoV-2 differs from other CoVs in features, such as: (1) S protein short anchor, (2) small ORFs’ distinctive amount and position, and (3) papain-like protease PLP^pro^’s one copy [2]. 

### SARS-CoV-2 Antigen Selection

SARS-CoV-2’s complete sequences have an impact on developing vaccines and antiviral agents [6]. Vaccine candidates target spike (S) and nucleocapsid proteins, including subunit and recombinant vector vaccines, as well as DNA vaccines [7]. Lucchese [18] proposed unique pentapeptides of SARS-CoV-2 that are considered as preventive measures against novel coronavirus (2019-nCoV). Pentapeptides are oligopeptidic sequences (*n* = 37) of the spike protein that are absent in humans [18]. Thus, both the S and nucleocapsid (N) proteins of SARS-CoV-2 are attractive immunogens for vaccine development [16]. The viral pentapeptides (*n* = 933) [18] are unfamiliar to the human immune system. Among these, the S glycoproteins (*n* = 107) utilize the receptor-binding domain (RBD) to bind to a human ACE2 receptor [18]. The ~1200 aa long S protein with the S1/S2 processing site exhibits different motifs among coronaviruses [19]. In 2019-nCoV, the RBD includes a receptor-binding motif (RBM), implicating eight conserved residues to contact ACE2 [30]. The S2-protein consists of the fusion amino acids (FP) and a second S2 cleavage site for virus entry [19,30]. The S2 subunit is highly conserved, thus it is a target for antiviral agents [23]. Interestingly, it is important for an ideal vaccine to induce highly potent neutralizing antibodies without inducing any disease-enhancing antibodies [31]. In fact, the antibody-dependent enhancement (ADE) of some viral infections can increase the virus cell entry and replication due to the presence of virus-specific antibodies [32]. However, the mechanisms of ADE still remain to be better studied and understood. Therefore, the identification of potent and effective viral epitopes and their safety assessment is an important aspect to be taken into consideration.

SARS-CoV-2’s RBD contains the neutralizing antigens, but does not have any linear immunodominant (ID) sites [27]. The RBD of SARS-CoV-2 showed a contrasting ID scene compared with its correlative in SARS-CoV. Vaccines against SARS-CoV-2 using the S linear antigenic epitopes instead of the entire S protein guarantee better safety [27]. Interestingly, ORF3b and ORF8 have no homology with SARS-CoV, thus will help with the construction of an adequate vaccine. Furthermore, Zhang et al. [27] reported that some protein-specific epitope titers outreached the microneutralization titers by several orders of magnitude. Additionally, because of the low level of neutralizing antibody measured in in the recovered patients, it is supposed to observe a compelling difference between specific and neutralizing antibodies. Authors also reported comparable antiviral activity followed by the immunization program, with epitopes compared to vaccination with the complete RBD fragment. This observation gives evidence that epitope-based vaccines can generate equivalent protection compared to subunit vaccines. Alternative vaccines based on DNA, expressing full-length S proteins or its fragments as well as nucleocapsids, have advantages (safety; neutralizing antibodies) and disadvantages (immune responses; TH2 cell-distortive immune response; delayed-type hypersensitivity) [18]. Thus, to increase the efficiency, these vaccines are administered with adjuvants [26]. Moreover, the inactivated whole-virus vaccines, containing CoV genetically attenuated in replication [33], are under study. The antiviral discovery processes includes the mRNA-based vaccines consisting of mRNAs encoding full-size S formulated on cationic lipid nanoparticles [33]. As far as it is known, vaccine development focuses on mRNA encoding S, E, M and N protein, all of which are potent in inducing the antigen-specific immune reactions [33].

## 4. Adenoviruses as a Promising Vaccine Adjuvant

A vaccine, in order to be effective and long-lasting, should induce protective immunity in a population towards a specific epitope/antigen. Such immunity is generated by the immunization process containing an element of the disease inducing agent (e.g., a killed or live attenuated form of the pathogen). Currently, many of the most successful and safe vaccines used attenuated variants of a target pathogen resulting in a mild infection able to produce long-lasting immunity [12]. Protein-based vaccines depend on the presence of adjuvants in order to induce both an innate and adaptive immune response and subsequently to generate protective immunological memory to the vaccine antigen, since proteins alone are often not very immunogenic [34]. Therefore, adjuvants are considered as additional key components of the vaccine for a successful immunization process within an individual by increasing the adaptive response to a vaccine [34].

One of such potent adjuvants can be the adenovirus vector [35,36,37,38,39,40,41,42,43,44,45,46,47]. In an effort to design vaccines rationally, different studies have tested replication-defective adenovirus (Ad) vectors as a vaccine platform [20,48]. Adenovirus vectors are promising as: (1) a vaccine vector because of their properties to stimulate the immune responses, and (2) an adjuvant because of their ability to stimulate the immune reaction [49]. On the top of adenovirus immunogenicity, adenoviral vectors have been extensively characterized showing: (1) a genome that can be easily modified by inserting exogenous transgenes of an interest, and (2) a relatively stable viral capsid. Moreover, adenovirus vectors can either encode for various exogenous antigens or can present those epitopes on the surface of the capsid, modulating a strong immune response to the specific antigens [50]. Furthermore, adenoviruses are able to activate various innate immune signalling pathways resulting in the secretion of a number of proinflammatory cytokines, paving the route for potent and adequate immune cell stimulation by inducing a strong adaptive humoral and cellular immune reaction which are translated into an immunological memory to the vaccine antigen.

Adenovirus-based vaccines can utilize replication-competent or replication-defective vectors, constructed by removing or replacing the E1A and E1B genes (early transcript 1A and 1B), thus erasing the replication features of vectors [51]. The viral E3 and E4 genes are often removed in order to diminish the virus clearance from the host [51]. Adenovirus itself works as a strong immune activator, enhancing the immunogenicity of the vaccine antigens, attracting antigen presenting cells to the side of infection, boosting T cell priming and inducing development of proinflammatory responses. Therefore, adenovirus properties of activating innate and adaptive immune responses helps develop immunity to the exogenous antigens and develop a protective immunity [52,53,54,55].

Adenoviruses exhibit several advantages over other viruses, such as: vaccinia virus (VV), lentivirus, retrovirus, adeno-associated virus (AAV) and herpesvirus. They are highly immunogenic and have an ability to induce potent innate and adaptive immune responses in a host. In contrast to a lentivirus or retrovirus, adenoviruses do not integrate the viral genomic DNA into the hosts’ chromosome, thus reducing the risk of mutagenesis [2,3]. In turn, AAVs are less pathogenic than adenoviruses, but on other hand big scale manufacturing of AAV is more complexed and challenging [4].

However, there are also some drawbacks of using adenoviruses (AdVs) as an adjuvant. This includes pre-existing immunity in humans leading to the production of neutralizing antibodies. Nevertheless, this limitation can be easily overcome by replacing the adenovirus hexon sequence or fiber knob domain (chimeric adenovirus) from a different serotype. Importantly, due to the versatility and diversity of adenovirus serotypes, they are valuable and preferable vectors for vaccine productions against a broad spectrum of pathogens [5,6]. AdVs have been classified into seven subtypes: A–G into 67 various serotypes [56]. This classification relies on similarities in genome homology, tropism and analyses of neutralizing antibodies against capsid antigen, phylogenetic assessment encoding protease, the hexon structure, and the viral DNA polymerase [57]. Because of the broad versatility and diversity of adenovirus subtypes, they are valuable and preferable vectors for vaccine productions against a broad spectrum of pathogens [57,58]. Many adenoviruses exhibit very low seroprevalence: AdV2, AdV26 and AdV35 are considered as promising candidates for a vaccine development [59]. Both adenoviruses AdV26 and AdV35 have been tested in clinical studies, where they have been proven to be well tolerated and safe. However, their efficacy and immunogenic potency is lower in comparison to the most well studied and used, AdV5. Since the neutralizing antibodies against various serotypes and T cells do not cross react with different subtypes, it gives broad possibilities for vaccine development, utilizing various vectors or their combinations [60,61].

In addition to their immune-stimulatory properties (both innate and adaptive), adenoviral-based vaccine vectors have been well characterized. Interestingly, their safety profile is well known and proven in many clinical studies [24,62,63]. Taking all this into account, using genetic engineering tools, it is doable to construct and produce Ad-based vectors to induce the strong and effective immune response towards the target vaccine antigen. This scientific rationale for the adenovirus-based vaccine construct strategy may contribute to a more effective vaccine, able to generate a specific immune response depending on the vaccine antigen.

## 5. Clinical Prospects on COVID-19 Vaccine Development

**Figure 1:**
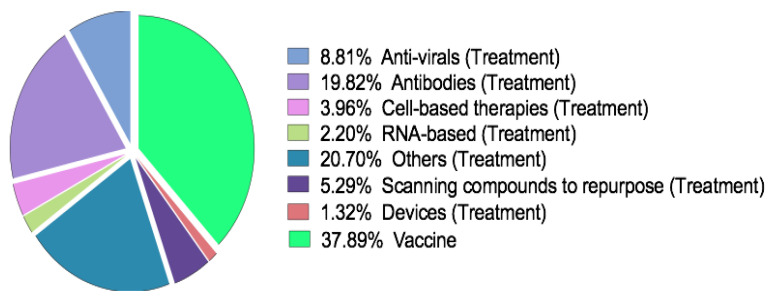
Coronavirus disease 19 (COVID-19) treatments and vaccines in a research pipeline with a percentage distribution.

Up to date, there is no available preventive vaccine against SARS-CoV-2 and no drug agent with proven clinical potency. However, there are multiple candidates that might be effective in prevention or treatment. Over 900 clinical trials have been registered at clinicaltrials.gov (27 April 2020) and over 1100 worldwide, including 123 trials with a EudraCT (27 April 2020) on COVID-19 covering both treatment and preventive approaches (Figure 1).

Although no vaccines are available on the market for both SARS and Middle East respiratory syndrome (MERS), considerable effort has been put into the vaccine development against these diseases, thus representing a valuable example to produce an effective vaccine for COVID-19. After the SARS epidemic in 2002–2003, the majority of vaccines [64] targeted the spike (S) glycoprotein of the virus [65]. Different vaccine strategies have been proposed based on a live-attenuated, inactivated virus, recombinant viral vectors, DNA, virus-like particles (VLPs) and soluble proteins [66]. Strategies based on recombinant viral vectors used genetically engineered viruses, which differ from the SARS-CoV and are able to express components of the SARS-CoV [67]. Currently, only vaccines based on an inactivated SARS virus, DNA and soluble proteins based on the SARS S glycoprotein, reached a clinical stage (phase I). As for SARS vaccines, most of them are based on the S glycoprotein as well. Vaccines based on inactivated and live attenuated viruses, recombinant viral vectors, nanoparticle DNA and soluble proteins have been developed and tested in animal models. Interestingly, so far only one DNA-based vaccine has been tested in phase I [68].

Currently there are at least two promising adenovirus-based vaccines against COVID-19 undergoing testing in clinical studies. The first one is based on the human replication-defective AdV5 vector (Ad5-nCoV) coding for the full-length S protein currently being tested in China (mid. April 2020, Phase I/II of the clinical study, NCT04313127, NCT04324606). Preclinical data on Ad5-nCoV showed the safety profile and the ability of the tested vaccine to generate strong immune responses in tested animal models. Another promising vaccine is based on Chimpanzee adenovirus (ChAdOx1) engineered in UK and is being tested in the phase I/II clinical trial (April 2020, NCT04324606) on 510 volunteers. The adenovirus vector is also replication-deficient and has been armed with genes encoding antigens in order to stimulate humoral and cytotoxic T-cells.

As reported by CEPI in April 2020, 115 total vaccine contestants are in early stages of development [69]. Selected clinical studies on a COVID-19 vaccine have been shortlisted and are presented in Table 1.

## 6. Conclusions

In the face of a current unprecedented world pandemic, urgent and rapid research focused on the production of clinically proven vaccines is in high demand.

The SARS-CoV-2 vaccination approach and technology should be supported and guided by a better understanding of the SARS pathogenesis in mankind, including the route of virus dissemination within the body. These aspects can support the generation of vaccines able to prevent and inhibit SARS-CoV-2 spread and, above all, infection of the vital and target organs. So far, unfortunately, we still have limited knowledge about SARS-CoV-2 and there are still a lot of open questions, in particular the immune reaction in response to the virus invasion, that is crucial for vaccine generation, remains unclear. All these issues need to be addressed in the near future for a successful vaccine development.

The potential of adenoviruses as vaccine vectors is attributed to their ability to drive a robust humoral and cellular adaptive immune response. Over recent years, there has been significant progress made in the field of adenovirus vector vaccine development. Currently we are entering an exciting stage, focused on the development of adenovirus-based vaccines, and the results from on-going clinical trials may contribute to improve our knowledge and develop an effective vaccine against COVID-19.

Persistent and constant international cooperation along with joint attempts are crucial to unravel the outstanding unanswered queries about the new SARS-CoV-2. Importantly, the cooperation on national and international levels encompasses: academics, governments and industries, which are imperative to limit dissemination of COVID-19 and to prevent future outbreaks.

## Figures and Tables

**Table 1:** Selected clinical studies on vaccine research against SARS coronavirus 2 (SARS-CoV-2).

Name	Description	Phase of the Trial	Number of Participants	Location	References
**INO-4800**	DNA plasmid	Phase I	40	USA	NCT04336410
**Ad5-nCoV**	Recombinant AdV 5	Phase I	108	China	NCT04313127
**Ad5-nCoV**	Recombinant AdV 5	Phase II	500	China	NCT04324606
**ChAdOx1 nCoV-19**	Adenovirus vector	Phase I, II	510	UK	NCT04276896
**LV-SMENP-DC**	Lentiviral vaccine, DCs modified with a lentiviral vector	Phase I, II	100	China	NCT04276896 [70]
**Covid-19/aAPC**	Lentiviral vector, pathogen specific artificial antigen presenting DCs	Phase I	100	China	NCT04299724 [70]
**mRNA-1273**	Lipid nanoparticle containing mRNA	Phase I	45	USA	NCT04283461
**rhACE2**	Recombinant ACE2 (angiotensin-converting enzyme 2)	-	24	China	NCT04287686
**Washed microbiota** **transplantation**	Washed microbiota transplantation	-	-	China	NCT04251767
**BCG Vaccination to Protect Healthcare Workers Against COVID-19 (BRACE)**	Bacillus Calmette–Guérin (BSG) vaccine	Phase 3	4170	Australia	NCT04327206
